# Descemet Membrane Endothelial Keratoplasty for Corneal Decompensation Secondary to Phakic Intraocular Lenses

**DOI:** 10.1155/2019/2038232

**Published:** 2019-10-27

**Authors:** Ester Fernández López, Cristina Peris Martínez

**Affiliations:** ^1^FISABIO Oftalmología Médica (FOM), Cornea and Anterior Segment Unit, Valencia, Spain; ^2^University of Valencia, Valencia, Spain

## Abstract

**Purpose:**

To describe the surgical technique and clinical outcomes of bilensectomy (pIOL explant and phacoemulsification), followed by DMEK performed for bullous keratopathy secondary to pIOL.

**Methods:**

Seven eyes of seven patients, who developed corneal decompensation after pIOL implantation, underwent bilensectomy followed by DMEK in a two-step procedure. Main outcome measures included uncorrected visual acuity (UCVA) and best-corrected visual acuity (BCVA), refraction, endothelial cell density (ECD) at 1, 3, 6, and 12 months, and intraoperative and postoperative complications.

**Results:**

DMEK was performed at a mean time of 9.83 ± 8.23 months after bilensectomy. BCVA (log MAR) improved in all eyes, increasing from 1.11 ± 0.78 preoperatively to 0.54 ± 0.21, 0.28 ± 0.23, 0.21 ± 0.21, and 0.17 ± 0.17 at 1, 3, 6, and 12 months after DMEK. One year after surgery, mean spherical equivalent and cylinder were −0.70 ± 0.92 D and −1.50 ± 0.54 D, respectively. ECD decreased by 62 ± 4%, 69 ± 4%, 74 ± 4%, and 77 ± 3% at 1, 3, 6, and 12 months after DMEK. There was one case of primary graft failure and no other postoperative complications.

**Conclusions:**

The two-step technique bilensectomy followed by DMEK is a feasible technique for the management of bullous keratopathy secondary to pIOL, providing a fast visual recovery with good visual and refractive results.

## 1. Introduction

Phakic intraocular lens (pIOL) implant is a well-established technique for the correction of moderate to high refractive errors, providing high quality of vision while preserving the corneal thickness. [1–4] In the past decades, there has been a continuous development in pIOL designs and surgical technique, nevertheless some postoperative complications may be encountered [5, 6] and their explantation might be necessary, especially with the first pIOL designs or an inadequate anatomy of the anterior chamber. In a large multicentric retrospective study of 240 explanted pIOL, the second main reason for pIOL explant after cataract formation was endothelial cell loss [7] being up to 15.97% of angle-supported pIOLs and 8.33% of iris-fixated pIOLs of the 240 cases. Some of these eyes may also present corneal decompensation, requiring additionally a corneal transplant to restore corneal transparency.

Descemet membrane endothelial keratoplasty (DMEK) has become the preferred technique for endothelial disorders, given its unprecedented visual and refractive outcomes and rapid visual recovery [8, 9]. While Fuchs dystrophy and pseudophakic bullous keratopathy continue to be the main indications for DMEK, technique standardization [10] and increased surgical experience have increased the spectrum of disorders suitable for DMEK such as failed corneal grafts, eyes with previous glaucoma surgeries, or vitrectomized eyes [11, 12]. Patients who have undergone refractive surgery including pIOL implantation are a subset of patients with high visual and refractive expectations, in a way that DMEK might offer them a better postoperative outcome compared with previous keratoplasty techniques.

There is currently limited information regarding the outcomes of DMEK in eyes with bullous keratopathy due to pIOLs. We describe a case series of patients with corneal decompensation secondary to different types of pIOLs managed with a two-step procedure bilensectomy (pIOL explant and phacoemulsification), followed by DMEK.

## 2. Materials and Methods

### 2.1. Patients

This is a retrospective case series of seven patients with corneal decompensation secondary to pIOL implant attending the Cornea Clinic at FISABIO Oftalmología Médica (FOM), Valencia, Spain. The medical records were reviewed and data collected included patient demographics, pIOL model, and time between implantation and explantation, preoperative and postoperative clinical examination findings, uncorrected visual acuity (UCVA), best-corrected visual acuity (BCVA), refraction, and endothelial cell density (ECD). All patients presented decreased endothelial cell counts with mild to moderate signs of corneal decompensation and cataract. Six patients underwent a two-step procedure bilensectomy (pIOL explant and phacoemulsification), followed by DMEK at least one month later. Quadruple procedure (bilensectomy, cataract surgery, intraocular lens implant, and DMEK) was performed in one of the patients. Preoperative and surgical data are summarized in Table 1.

### 2.2. Phakic Intraocular Lens Explant and Phacoemulsification

Phacoemulsification was performed under peribulbar anesthesia in all patients. A superior conjunctival peritomy and a 6.5 mm scleral tunnel were made with a 15° slit knife and crescent blade into clear cornea. One or two side port incisions were created, depending on the pIOL model to aid explantation, and sodium chondroitin sulfate-sodium hyaluronate (Viscoat®) was injected into the anterior chamber. The anterior chamber was entered with a 2.75 mm surgical blade. The pIOL was released from the angle or iris, rotated, and explanted through the scleral wound. Before phacoemulsification, the scleral incision was sutured with a 10/0 nylon leaving a 2.75 mm entrance for the phacoemulsification tip. Phacoemulsification was performed in a standard fashion with the Infiniti System (Alcon), and a spherical intraocular lens was implanted in the capsular bag in six cases (one case was left aphakic because IOL calculation resulted in a 0 D power IOL). Intraocular lens (IOL) power calculation was performed with the IOLMaster (V.408; Carl Zeiss Meditec, Jena, Germany) using the SRK-T formula, except in two cases where ultrasound biometry (A-Scan DGH 6000, DGH Technology, Inc.) was used as readings were not possible with the IOLMaster due to corneal decompensation. Target refraction was between −0.50 and −1.0 D in all cases. Intracameral cefuroxime and subconjunctival betamethasone were injected at the end of the case. Patients followed a postoperative regime of 0.3% ofloxacin drops three times a day for 1 week, 0.1% dexamethasone drops with a tapering regime during at least 1 month, and diclofenac drops three times a day for 1 month.

### 2.3. Descemet Membrane Endothelial Keratoplasty Surgery

DMEK grafts were prepared by the surgeon immediately prior to transplantation or the day before using Melles technique [13]. Corneal-scleral buttons were mounted endothelial side up on a Barron Vacuum Corneal Punch (Katena, Inc., Denville, NJ). The Descemet membrane was loosened from the scleral spur with a hockey stick knife and then stripped from the corneal stroma with McPherson forceps. The Descemet membrane was cut with a 7.0 to 8.0 mm punch. DMEK was performed under peribulbar anaesthesia using the Melles standardized no-touch technique [10] with the modification of using gas instead of air as a tamponade. All patients had previous iridectomies during the pIOL implant. A 2.75 mm corneal tunnel incision was made at the limbus at 12 o'clock position and 3 side ports. Descemetorhexis was performed under air using a reversed Sinskey hook. The donor Descemet roll was stained for 3 minutes with 0.06% trypan blue (Vision Blue, DORC), transferred to a DMEK glass pipette (DORC International BV), and introduced through the main incision. After checking correct orientation with the Moutsouris sign [10], the graft was unfolded with the no-touch technique with fluid and air. 20% concentrated sulfur hexafluoride gas was injected to position the tissue against the posterior stroma, leaving a full tamponade for one hour and performing fluid-gas exchange leaving about a 1 mm peripheral meniscus free of gas. No patient required further gas evacuation or mydriatic drops to avoid pupillary block after surgery.

Postoperative topical treatment consisted of 0.3% ofloxacin drops four times a day for 2 weeks, 1% prednisolone drops four times a day for 1 month, followed by 0.1% fluorometholone drops four times a day tapered down to one drop a day one year after surgery. Patients were examined on day 1, week 1, and months 1, 3, 6, and 12 after surgery. Slit-lamp examination, Visante OCT (Carl Zeiss Meditec, Jena, Germany), and specular microscopy (SP-3000P, Topcon Corporation, Tokyo, Japan) were performed during each visit.

## 3. Results

In this case series, we report the results of seven patients with corneal decompensation secondary to pIOL who underwent pIOL explantation, cataract surgery, and DMEK. The mean age of the patients was 55 ± 6.76 years (range: 45–63 years). Preoperatively, all patients had severe endothelial cell loss and corneal decompensation ranging from Descemet folds (3 patients) to Descemet folds and subepithelial bullae (4 patients). Mild to moderate cataractous changes were present in all eyes. Preoperative BCVA ranged from 20/2000 (2 log MAR) to 20/33 (0.2 log MAR). The patients had no other concomitant ocular diseases besides high myopia, which in one eye (patient 2) led to amblyopia and patient 4 who had a dome-shaped macula. One patient (patient 5) had pIOL subluxation secondary to trauma and underwent pIOL refixation followed by pIOL explant in another clinic.

The explanted pIOL models were Artisan iris-fixated lens (Ophtec BV, Groningen, The Netherlands) in four eyes, I-Care (Corneal, Pringy, France) in two eyes, and GBR/Vivarte (Zeiss Meditec, Jena, Germany) in one eye. The GBR/Vivarte and I-Care lenses had already been withdrawn from the market in 2006 and 2008, respectively, because of safety concerns related to endothelial cell loss. The mean time between pIOL implantation and explantation was 14.17 ± 4.14 years (range: 7–21 years). Six patients underwent the two-step procedure bilensectomy followed by DMEK. One patient had a quadruple procedure, DMEK being performed on the same day than pIOL explant and cataract surgery. Patients 5 and 7 had the pIOL explanted and cataract surgery in another hospital. DMEK was performed at a mean time of 9.83 ± 8.23 months (range: 1–26 months) after pIOL explantation and phacoemulsification. Baseline characteristics and surgical data are summarized in Table 1.

The endothelium-Descemet membrane could be stripped successfully in all cases, although the punch size had to be decreased to 7 mm in one case to avoid peripheral tears. DMEK was uneventful in all eyes, although surgical time of DMEK in the quadruple procedure was considerably longer. All patients except for patient 6 reached the 12-month postoperative visit. UCVA, BCVA, refraction, and endothelial cell densities are detailed in Table 2.

**Figure 1:**
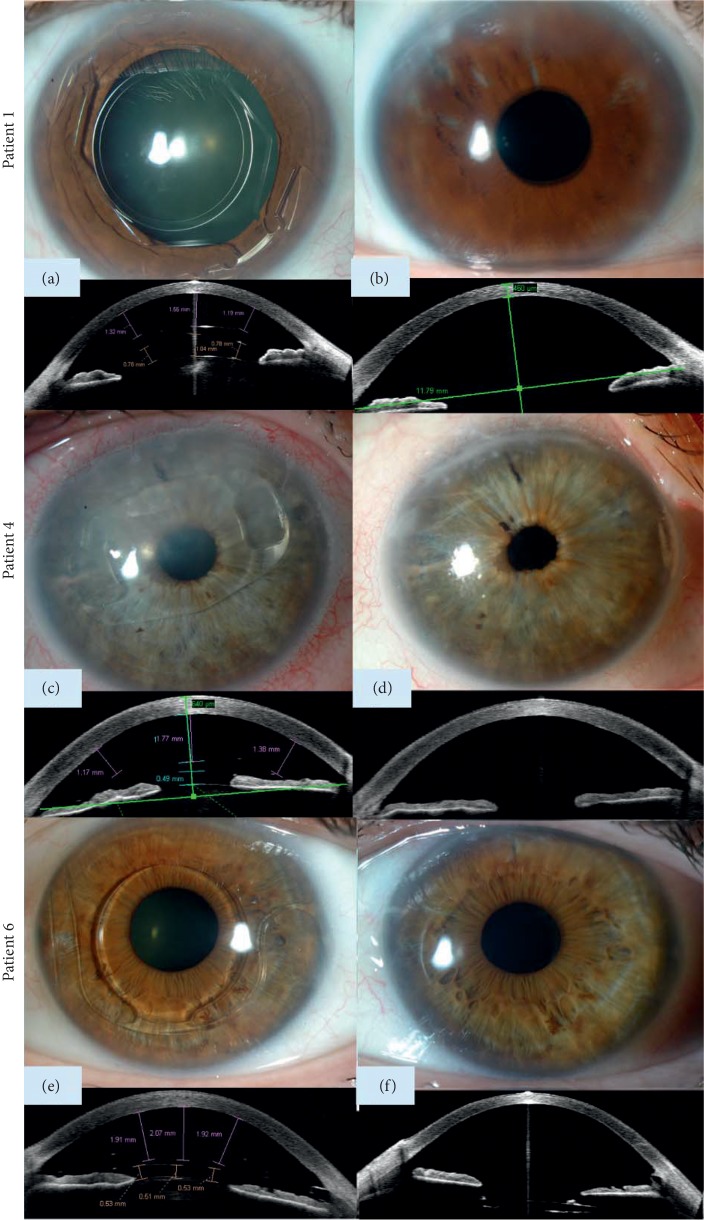
Preoperative and postoperative slit-lamp images and Visante OCTs of three of the cases: (a, b) patient 1 with I-Care phakic intraocular lens (pIOL), (c, d) patient 4 with Artisan pIOL, (e, f) and patient 6 with GBR/Vivarte pIOL.

**Figure 2:**
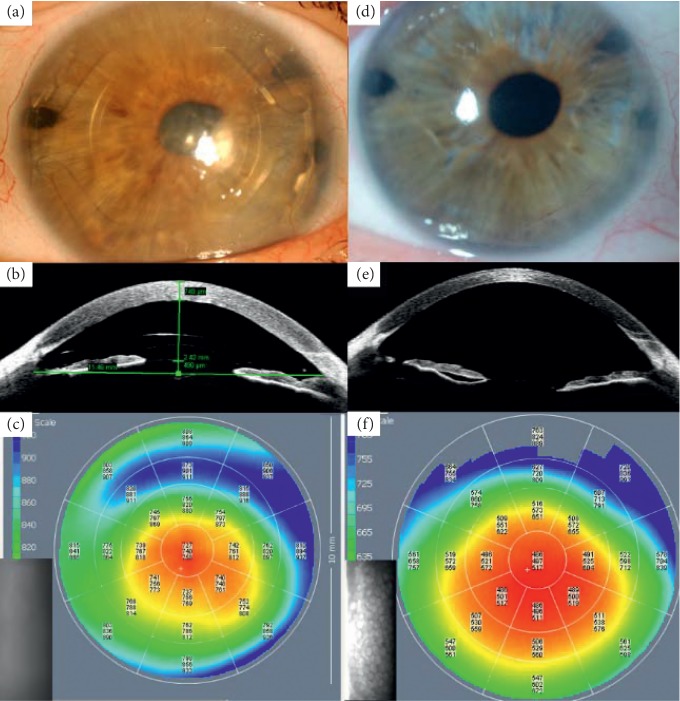
A 59-year-old woman (patient 2) with I-Care pIOL implanted for 21 years underwent bilensectomy followed by Descemet membrane endothelial keratoplasty (DMEK) 1 month later. (a–c) Preoperative slit-lamp and Visante OCT images revealed a decompensated cornea with increased central corneal pachymetry of 740 *μ*m and uncountable central endothelial cells on the specular microscope. Preoperative uncorrected visual acuity (UCVA) and best-corrected visual acuity (BCVA) were 20/2000 (counting fingers). (d–f) Six months postoperatively, there was a resolution of the corneal edema with improved central pachymetry to 497 *μ*m and endothelial cell density of 870 cells/mm^2^. UCVA increased to 20/44 (0.45) and BCVA to 20/30 (0.65), being an amblyopic eye.

BCVA improved in all patients, increasing from 1.11 ± 0.78 log MAR preoperatively to 0.54 ± 0.21 log MAR, 0.28 ± 0.23 log MAR, 0.21 ± 0.21 log MAR, and 0.17 ± 0.17 log MAR at 1, 3, 6, and 12 months, respectively, after DMEK. One year after surgery, mean spherical equivalent and cylinder were −0.70 ± 0.92 D and −1.50 ± 0.54 D, respectively. ECD decreased from 2975.71 ± 326.01 cells/mm^2^ in donor corneas before DMEK preparation to 1131.50 ± 217.22 cells/mm^2^ (62 ± 4% loss), 927.00 ± 171.47 cells/mm^2^ (69 ± 4% loss), 771.33 ± 105.56 cells/mm^2^ (74 ± 4% loss), and 717.50 ± 43.91 cells/mm^2^ (77 ± 3% loss) at 1, 3, 6, and 12 months after DMEK, respectively. Figures 1 and 2 demonstrate clinical pictures of 4 of the cases.

All grafts were completely attached after surgery without requiring any rebubbling procedures. No cases of intraocular pressure spikes, pupillary block glaucoma, or graft rejection were observed during the follow-up period. There was one case of primary graft failure (patient 4), who underwent bilensectomy and DMEK on the same day with an increased surgical time for DMEK and smaller DMEK graft size, both factors probably being the cause for primary graft failure. Two eyes underwent YAG capsulotomy during the follow-up period (patients 2 and 5).

## 4. Discussion

Severe endothelial cell loss is a rare but severe complication after pIOL implant, which can be challenging to manage for the corneal surgeon, as it implies two clinical issues: first, the high refractive error and anisometropia if only the pIOL is explanted; second, the possibility of requiring a corneal transplant if the cornea further decompensates. Besides, this subset of patients consists of relatively young patients who underwent refractive surgery with no previous ocular comorbidities and therefore have high visual and refractive demands. Only a few case reports and case series have described the management of corneal decompensation in patients with pIOL [14–18]. To the best of our knowledge, this is the first case series describing the two-step procedure, bilensectomy followed by DMEK for the management of this entity, with its clinical outcomes.

pIOL explant usually needs to be followed by cataract surgery or clear lens extraction in some cases in order to maintain the refractive benefits obtained with the pIOL in these patients and avoid postoperative anisometropia if only the pIOL is explanted. Besides, pIOL explantation and/or a subsequent keratoplasty might pose an additional cataractogenic risk, especially considering they are highly myopes with an increased risk of early cataract formation. In our case series, all patients presented at least mild nuclear sclerotic changes and no clear lens extractions were necessary. pIOL explantation can be performed through a limbal or scleral incision. In our cases, we preferred the sclera as the incision site to avoid a large corneal incision that could interfere with DMEK or increase the postoperative refractive error. In our patients, the postoperative mean spherical equivalent and cylinder were −0.70 ± 0.92 D and −1.50 ± 0.54 D, respectively, and only 2 patients had a postoperative astigmatism of more than 2 D. One of them (patient 5) who had pIOL subluxation secondary to trauma and underwent pIOL explant in another clinic and another patient with high preoperative astigmatism where a spherical lens was implanted to avoid possible refractive surprises with a toric intraocular lens, given the advanced bullous keratopathy.

Previous case reports and case series have described mainly penetrating keratoplasty [7] and Descemet stripping automated endothelial keratoplasty [14–16, 18] in cases of bullous keratopathy secondary to pIOLs. Only Liarakos et al. [17] report DMEK surgery in 8 eyes with previous pIOLs (7 eyes which had the pIOL explanted elsewhere but crystalline lens left in situ and 1 eye where the pIOL was not explanted before DMEK). DMEK has become the procedure of choice for treating corneal endothelial disorders as it provides a faster visual rehabilitation, an optimal visual quality comparable to a normal cornea, and a higher refractive predictability [19, 20]. Therefore, we believe for this subset of patients with bullous keratopathy secondary to pIOL, DMEK might be the best choice, considering their younger age and high visual and refractive demands.

In this series, DMEK proved feasible in all eyes, with no intraoperative or early postoperative complications. The ECD decrease was slightly higher during the first year compared to other series but the grafts remained clear and stable during the follow-up period. BCVA improved in all eyes, being 20/32 or above in 5 patients (71.43%), 12 months after surgery. Moreover, 3 eyes (42.86%) already achieved 20/32 BCVA or above, 3 months postoperatively, representing a fast visual rehabilitation, compared to other keratoplasty techniques. Regarding the two eyes with lower vision, one was an amblyopic eye (patient 2) and patient 4 (who underwent the quadruple procedure), besides a significant myopic retinopathy with dome-shaped macula had a primary graft failure. Although reasonable UCVA outcomes were achieved, spectacle or contact lens correction should also be anticipated and patients should be appropriately counseled preoperatively.

DMEK could have also been combined with simultaneous pIOL explantation and cataract surgery, the so-called quadruple procedure. Quadruple procedure has been previously described with Descemet stripping automated endothelial keratoplasty [15, 18]. Although it can also be feasible with DMEK, we prefer the two-step procedure because of several reasons. Although it was not the case in this case series, cases with severe endothelial decrease secondary to pIOL might not evolve to bullous keratopathy after pIOL explantation; hence, DMEK might not be mandatory in all cases. Moreover, quadruple procedures might increase intraoperative and early postoperative complications. For instance, in some eyes with long-standing pIOLs, releasing the haptics may require cutting adhesions that might lead to intraoperative bleeding, interfering with DMEK surgery. In our case series, the only quadruple procedure had primary graft failure; although a larger prospective study would be needed to confirm the clinical outcomes, one may consider sequential procedures preferable. This has, however, to be thoroughly discussed with the patients, as sequential procedures may also have some disadvantages, including an additional surgery and a decrease in visual acuity and increase in discomfort while awaiting the corneal transplant.

## 5. Conclusions

In summary, DMEK performed after bilensectomy appears to be a feasible technique for the management of pIOL corneal decompensation, providing a fast visual recovery with good visual and refractive results. Further studies with larger patient cohorts and follow-up are desirable to confirm the results of this technique.

## Figures and Tables

**Table 1:** Preoperative data and surgical details of the seven patients.

Patient	Age/Sex	Explanted pIOL model (time inside eye—years)	Cataract (LOCS scale)	Corneal status	Pre-op BCVA Snellen (log MAR)	Pre-op ECD (cells/mm^2^)	Surgery performed
1	63/F	I-Care (13 y)	N2C2P0	Descemet folds	20/50 (0, 4)	580	Bilensectomy, 8 mm DMEK 10 months later
2	59/F	I-Care (21 y)	N1C2P0	Descemet folds and bullae	20/2000 (2)	No readings	Bilensectomy, 7.75 mm DMEK 1 month later
3	49/M	Artisan (16 y)	N1C0P0	Descemet folds and bullae	20/2000 (2)	857	Bilensectomy, 8 mm DMEK 5 months later
4	63/F	Artisan (14 y)	N2C2P0	Descemet folds and bullae	20/100 (0.7)	No readings	Quadruple procedure: bilensectomy and 7 mm DMEK
5	45/F	Artisan (7 y)	—	Descemet folds and bullae	20/2000 (2)	No readings	Bilensectomy performed elsewhere, 8 mm DMEK 13 months later
6	49/F	GBR/Vivarte (−)	N1C1P0	Descemet folds	20/33 (0.2)	754	Bilensectomy, 7.25 mm DMEK 4 months later
7	57/F	Artisan (14 y)	—	Descemet folds	20/63 (0.5)	No readings	Bilensectomy performed elsewhere, 8 mm DMEK 26 months later

pIOL: phakic intraocular lens; M: male; F: female; BCVA: best-corrected visual acuity; ECD: endothelial cell density; Descemet membrane endothelial keratoplasty (DMEK).

**Table 2:** Preoperative and postoperative outcomes of the seven patients.

Patient	UCVA Snellen (log MAR)					BCVA Snellen (log MAR)					Refraction 12 Mo	ECD (cells/mm^2^)				
	Pre-op	1 Mo	3 Mo	6 Mo	12 Mo	Pre-op	1 Mo	3 Mo	6 Mo	12 Mo		Donor	1 Mo	3 Mo	6 Mo	12 Mo
1	—	20/32 (0.2)	20/32 (0.2)	—	—	20/50 (0.4)	—	20/25 (0.1)	20/25 (0.1)	20/20 (0)	1.25 (−1.75 × 90°)	3125	1032 (67)	792 (75)	681 (78)	—

2	20/2000 (2)	20/125 (0.8)	20/63 (0.5)	20/40 (0.3)	20/32 (0.2)	20/2000 (2)	20/63 (0.5)	20/50 (0.4)	20/32 (0.2)	20/32 (0.2)	0 (−0.75 × 65°)	2777	—	—	870 (69)	785 (72)

3	20/2000 (2)	20/63 (0.5)	20/63 (0.5)	20/63 (0.5)	20/40 (0.3)	20/2000 (2)	—	—	20/25 (0.1)	20/32 (0.2)	0 (−1 × 40°)	3012	1308 (57)	973 (68)	932 (69)	713 (76)

4	20/100 (0.7)	20/200 (1)	20/100 (0.7)	20/125 (0.8)	20/100 (0.7)	20/100 (0.7)	20/100 (0.7)	20/100 (0.7)	20/100 (0.7)	20/63 (0.5)	−1.75 (1.25 × 65°)	2976	—	No readings	No readings	No readings

5	20/2000 (2)	20/400 (1.3)	20/45 (0.35)	20/50 (0.5)	20/40 (0.3)	20/2000 (2)	20/125 (0.8)	20/40 (0.3)	20/32 (0.2)	20/25 (0.1)	−0.25 (−2.25 × 105)	3225	No readings	920 (72)	641 (80)	662 (80)

6	20/63 (0.5)	20/63 (0.5)	20/40 (0.3)	—	—	20/33 (0.2)	20/63 (0.5)	20/32 (0.2)	20/32 (0.2)	—	−1.5 (6 Mo)	2314	824 (64)	728 (69)	700 (70)	—

7	20/63 (0.5)	20/32 (0.2)	—	—	—	20/63 (0.5)	20/32 (0.2)	20/20 (0)	20/20 (0)	20/20 (0)	1 (−2.00 × 95°)	3401	1362 (60)	1222 (64)	804 (76)	710 (79)

UCVA: uncorrected visual acuity; BCVA: best-corrected visual acuity; ECD: endothelial cell density.

## Data Availability

The data used to support the findings of this study are included within the article.
